# *Schistosoma mansoni* and other intestinal parasitic infections in schoolchildren and vervet monkeys in Lake Ziway area, Ethiopia

**DOI:** 10.1186/s13104-018-3248-2

**Published:** 2018-02-20

**Authors:** Dejene Teklemariam, Mengistu Legesse, Abraham Degarege, Song Liang, Berhanu Erko

**Affiliations:** 1 Hawassa College of Health Sciences, P.O. Box 84, Hawassa, Ethiopia; 2 Aklilu Lemma Institute of Pathobiology, Addis Ababa University, Addis Ababa, Ethiopia; 3 Department of Epidemiology, Robert Stemple College of Public Health, Florida International University, Miami, USA; 4 Department of Environmental and Global Health, College of Public Health and Health Professions, University of Florida, Gainesville, FL 32610 USA; 5 Emerging Pathogens Institute, University of Florida, Gainesville, FL 32610 USA

**Keywords:** *Schistosoma mansoni*, Intestinal parasites, Vervet monkeys, Zoonosis, Transmission, Ethiopia

## Abstract

**Objective:**

To assess *Schistosoma mansoni* and other intestinal parasitic infections in schoolchildren and vervet monkeys (*Chlorocebus aethiops*) in Bochessa Village, Ziway, Ethiopia.

**Results:**

Fecal specimens from selected schoolchildren and droppings of the vervet monkeys were collected and microscopically examined for intestinal parasites using the Kato-Katz thick smear and formol-ether concentration techniques. The prevalences of *S. mansoni*, *Trichuris trichiura*, *Ascaris lumbricoides*, *Enterobius vermicularis*, hookworms, *Hymenolepis nana* and *Taenia* species among the children were 35.7, 26.9, 24.1, 2.1, 2.1, 1.07 and 2.1%, respectively (by Kato-Katz) and 39.3, 36.1, 35.6, 2.9, 10.0, 4.3, and 2.9%, respectively (by formol-ether concentration). Prevalence of *S. mansoni* in vervet monkeys ranged from 10 to 20%. *B. pfeifferi* snails were exposed to *S. mansoni* miracidia from vervet origin, shed cercariae were then used to infect lab-bred albino mice. Adult worms were harvested from the mice 5 weeks post-exposure to cercariae to establish the schistosome life cycle and confirm the infection in the vervet monkeys. The natural infection of *S. mansoni* in vervet monkeys suggests that the non-human primate is likely to be implicated in the local transmission of schistosomiasis. Further epidemiological and molecular studies are needed to fully elucidate zoonotic role of non-human primate in the area.

## Introduction

Intestinal parasites cause one of the most prevalent infections in humans in developing countries [1]. Over 140 zoonotic parasites are known to be shared between humans and animals [2, 3]. Although spatial separation exists between human and non-human primates (NHPs) and other animals, indirect contact via environmental media such as air, soil and water, makes the human-NHPs linkage potentially important in the transmission of certain zoonoses [4, 5].

Humans are primary definitive host for *S. mansoni* [6] while the parasite also naturally infects other animals [7–12], suggesting that the animals (non-human) may play a role in the epidemiology of the disease. In most parts of Ethiopia, humans are the definitive host involved in the life cycle of schistosomiasis without reservoir hosts. Nevertheless, some studies conducted in the Rift Valley of Ethiopia suggested that NHPs were involved in the transmission of *S. mansoni* [11, 13].

Besides schistosome, studies also suggested that NHPs are naturally infected with protozoa and other intestinal helminths [13]. *Schistosoma* and other helminth species that can infect both monkeys and humans may mix to produce zoonotic hybrids capable of infecting both organisms. This will influence the transmission dynamics and morbidity due to helminth species in endemic regions where monkeys and human shares same habitats.

In Lake Ziway area of Ethiopia, although human infections with *S. mansoni* and other intestinal parasites were reported to be prevalent historically [14], the present status remains unclear. Additionally, free ranging monkeys are also common in the area but natural infections with these parasites have not been assessed. The objective of this study was to determine the prevalence of schistosomiasis and other intestinal helminthiasis among school children and free ranging vervet monkeys in Bochessa area, Ziway, Ethiopia. The results would provide important information for next step work to understand the roles of vervet monkeys in the transmission of schistosomiasis and other intestinal helminthiasis.

## Main text

### Methods

#### Study area and population

**Fig. 1:**
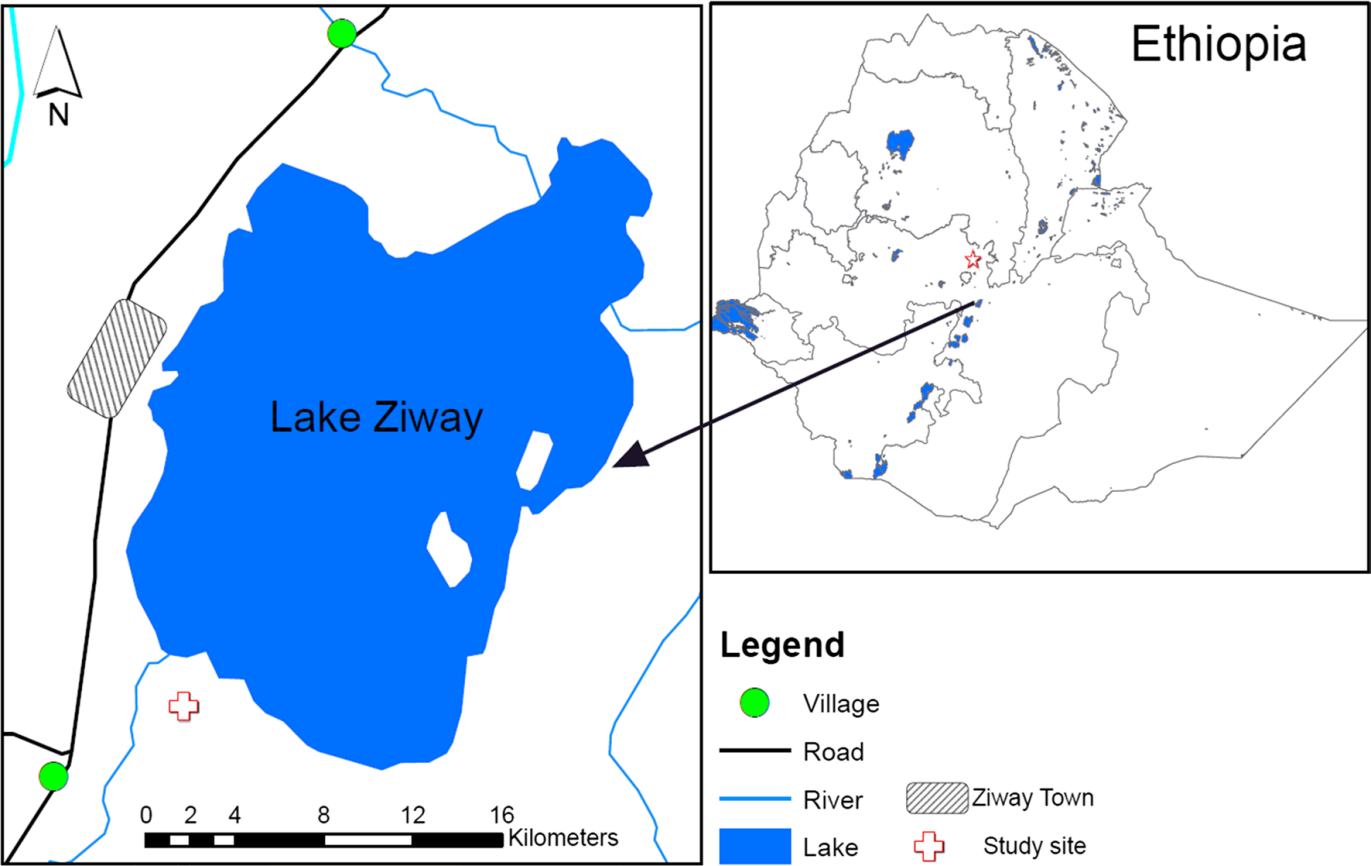
Map showing the study site near Lake Ziway, Ethiopia

The study was conducted in Bochessa Village, located about 5 km to the southeast of Ziway Town on the south shore of Lake Ziway (Fig. 1). Ziway Town (7°56′N 38°43′E, 1643-meter elevation) is located about 160 km to the south of Addis Ababa. The study participants were schoolchildren enrolled at Bochessa Elementary School in Bochessa Village and free ranging vervet monkeys (*Chlorocebus aethiops*) in the vicinity of the school.

#### Study design, stool specimen collection and microscopy

Cross-sectional parasitological surveys involving school children in Bochessa Elementary School and free ranging vervet monkeys were conducted in Bochessa Village, Ziway, from February to April 2011. A total of 280 schoolchildren were recruited in the study. The study participants were selected by systematic random sampling using school roll as a sampling frame. Before specimen collection, demographic information such as name, sex and age of each study participant were recorded. Then the study participants were provided with plastic sheet, toilet paper and applicator stick and requested to bring sizable stool samples. A portion of the stool sample was immediately processed using the Kato-Katz method (single slide, 41.7 mg template) while about 2-g of samples were preserved in labeled screw capped vials pre-filled with 10% formalin and later processed for microscopic examination by formol-ether concentration [15]. The Kato-Katz slides were examined for hookworms within 1 h of preparation while examination for the rest of helminths was done after 24 h of slide preparation at the Ziway Health Center. The formalin preserved specimens were transported and examined at the Aklilu Lemma Institute of Pathobiology. All the specimens were systematically examined under light microscope by experienced laboratory technicians.

#### Collection of faecal droppings of vervet monkeys and microscopy

Faecal droppings of 2 troops (~ 20 in each troop) of free ranging vervet monkeys were collected early in the morning before the vervet monkeys ascended from trees. One troop ranged over the immediate lake shore while the other troop ranged close to the Kobo Village. Both troops had contact with swamps and the lake water. Distinct faecal droppings of each troop were carefully picked up while fresh in the morning. Faecal droppings were collected in two separate vials, one with 10% formalin and the other with normal saline for the determination of the prevalence of schistosomiasis and infectivity, respectively. The faecal droppings preserved in 10% formalin were processed for microscopic examination by concentration method.

#### Hatching and infectivity test

The faecal droppings in the saline were pooled, washed several times in freshwater and passed through tiered sieves of varying mesh sizes. *S. mansoni* eggs collected on the sieve were placed in distilled water and exposed to light to stimulate hatching of miracidia to determine the viability of the eggs. Lab-bred *Biomphalaria pfeifferi* were exposed to *S. mansoni* miracidia from monkey origin to see the infectivity of the miracidia to the snails. After patent period (after 4 weeks) of the infected snails, ten mice were exposed en masse to cercariae shed by the infected *B. pfeifferi* snails to establish the lifecycle of the parasite.

The mice exposed to the cercariae were maintained, fed and humanely treated as per the guidelines on the care and use of animals for scientific purposes. After 5 weeks of post-exposure to the cercariae, each mouse received intraperitoneally 0.3 mL of anesthetic-anticoagulant solution (pentobarbital sodium and heparin). The mice were then sacrificed and adult *S. mansoni* were harvested from blood vessels around the liver and intestine.

#### Data analysis

The data were entered into Microsoft Office Excel 2007, cleaned and checked and analyzed in STATA. The prevalence of infection was computed by dividing number positive by number examined and expressed as percentage. Two sample test of proportion using z-statistics was performed to compare the prevalence of different helminth species in school children as determined by the Kato Katz and formol-ether technique. A 95% confidence interval was also determined for the differences in the prevalence of the different helminth species infection by the two methods.

### Results

#### Prevalences of helminths in schoolchildren

The prevalences of intestinal helminth infections among 280 schoolchildren a s determined by the Kato-Katz technique and formol-ether concentration are presented in Table 1. Except for *S. stercoralis*, which was not detected by the Kato-Katz, the two methods detected nine intestinal helminths in school children. The prevalences of *Trichuris trichiura*, *Ascaris lumbricoides*, hookworms and *Hymenolepis nana* as determined by formol-ether concentration were significantly greater than the prevalences of these infections as determined by Kato-Katz method. The formol-ether concentration technique also estimated greater percentage of children with *S. mansoni*, *Enterobius vermicularis s*and *Taenia* species infection as compared to the Kato Katz technique. However, these differences were not significant.

**Table 1:** Prevalence (%) of *S. mansoni* and other intestinal helminth infections among schoolchildren in Bochessa Primary School children, Ziway, 2011

Helminth species	Kato-Katz	Formol-ether	Prevalence difference (95% CI)	P value for the difference
*S. mansoni*	35.7	39.3	3.6 (− 0.4, 11.6)	0.3789
*T*. *trichiura*	26.9	36.1	9.2 (1.5, 16.9)	0.0191
*A. lumbricoides*	24.1	35.6	11.5 (4.0, 19.0)	0.0029
*E. vermicularis*	2.1	2.9	0.8 (− 0.02, 0.33)	0.544
Hookworms	2.1	10.0	7.9 (4.0, 11.8)	< 0.001
*S. stercoralis*	–	1.4	–	–
*H. nana*	1.07	4.3	3.2 (0.1, 5.9)	0.0181
*Taenia* spp.	2.1	2.9	0.8 (− 0.2, 3.4)	0.544

#### Helminth infections in monkeys

Microscopic examination of faecal droppings of free ranging vervet monkeys on the immediate lake shore and Kobo areas around the village showed of 20 and 10% of *S. mansoni* prevalence, respectively, as determined by concentration method. Other intestinal parasites detected in the pooled faecal droppings of the monkeys include *T. trichiura*, *A. lumbricoides*, hookworms, *Strongyloides* spp., *E. histolytica/dispar* and *E. coli*.

Lab-bred *B. pfeifferi* exposed to *S. mansoni* miracidia from monkey droppings shed cercariae after 4 weeks of exposure. Out of 10 mice exposed en masse to the cercariae 5 died before patency period and the remaining 5 mice survived and sacrificed after 5 weeks of exposure. A total of 97 adult male *S*. *mansoni* worms were harvested from the five mice.

### Discussion

Zoonoses have been receiving increased attention in the past decades because many pathogens of animal origin have been passing species barriers and causing serious health problems to humans, and vice versa. It is estimated that 75% of human pathogens are zoonotic, implying the great importance of pathogens of animal origin [16]. We conducted a preliminary study aimed to determine the occurrence and prevalence of *S. mansoni* and other intestinal parasitic infections in vervet monkeys (*Chlorocebus aethiops*) and schoolchildren in Bochessa Primary School in Bochessa Village, Ziway, Ethiopia. The findings showed that the vervet monkeys and school children share parasites including *S. mansoni*, *T. trichiura*, *A. lumbricoides*, hookworms, and *Strongyloides* species.

The most common intestinal parasitic infections identified among schoolchildren in Ziway were *S. mansoni* (35.7%), *T. trichiura* (26.9%), and *A. lumbricoides* (24.1%) as determined by the Kato-Katz method. In one study conducted in a rural community in southeast of Lake Langano in Ethiopia, Legesse and Erko [13] reported the prevalence of infection of 30.6, 18.8, and 8.2% respectively for *S. mansoni*, *T. trichiura*, and *A. lumbricoides*. In Wondo Genet, southern Ethiopia, Erko and Medhin [17] reported prevalence of 30, 88 and 77%, respectively for *S. mansoni*, *T. trichiura*, and *A. lumbricoides* infections. Such variations among study areas are expected in view of variations in ecological and climatic factors of the study areas among other things.

Overall, the formol-ether concentration technique identified higher numbers of *T. trichiura*, *A. lumbricoides*, hookworms and *Hymenolepis nana* infections than those by the single Kato Katz method. A study also showed higher sensitivity of the formol ether technique than the Kato Katz technique for the diagnosis of *T. trichiura*, *A. lumbricoides*, hookworms [18]. This difference in the prevalence of *T. trichiura*, *A. lumbricoides*, hookworms and *Hymenolepis nana* determined by the formol ether technique and the Kato Katz method could be due to the differences in the size of stool sample examined; ∼ 2 g in formol ether concentration vs 41.7 mg in Kato Katz method. Increased amount of the stool sample examined through the formol ether techniques improves detection of light intensity soil transmitted helminth infection which can be missed by the Kato Katz method [19]. Examining two or more Kato Katz thick smears from a single or different stool samples would help improve the performance of the Kato-Katz method for the diagnosis STHs [20, 21].

In the present study, the prevalence of *S. mansoni* infection in vervet monkeys (*Chlorocebus aethiops*, formerly referred to as *Cercopithecus aethiops*) was as high as 20%. Several study conducted in different parts of the Rift Valley also reported natural infections of *S. mansoni* in NHPs. Fuller et al. [7] recovered *S. mansoni* eggs and adult worms in baboons and vervet monkeys in Omo National Park in southwest Ethiopia. In a survey conducted in Kime area in the eastern shore of Lake Langano, Erko et al. [11] reported *S. mansoni* prevalence as high as 26.2% in the baboons. Legesse and Erko [13] also reported *S. mansoni* infection prevalence of 20.3% in baboons from same study area previously surveyed by Erko et al. [11].

In the present study, the species richness and diversity of gastrointestinal parasites observed in the vervet monkey were lower compared with species richness and diversity reported in NHPs in other parts of Africa. We only identified five helminth and one protozoan parasites. In Kenya, Mbora et al. [22] identified 21 gastrointestinal parasites in four groups of the Tana River mangabey (*Cercocebus galeritus*). Kouassi et al. [23] reported 9 protozoa and 14 helminths in seven non-human primate species in Taï National Park, Côte d’Ivoire. Kooriyama et al. [24] reported thirteen parasite species in five primates in Mahale National Park of Tanzania, and Gillespie et al. [25] reported 12 species of gastro-intestinal parasites in 3 colobus monkey species. Variations in the species richness and diversity of gastrointestinal parasites between different studies are attributed to differences in the number and species of NHPs surveyed differences in carpological methods employed as well as differences in ecology of the primates.

Identification and consideration of reservoir animals including NHPs in a control program is likely to be crucial for interruption of schistosomiasis transmission and elimination of the disease in a short-range because these animals may constitute important source of infection for humans in endemic areas. Hence, the argument of Angeles et al. [26] that elimination guidelines for schistosomiasis should include surveillance of the animal reservoirs is likely important.

In conclusion, the results of the study show that *S. mansoni*, *T. trichiura*, *A. lumbricoides*, hookworms, and *Strongyloides* species occur in school children and vervet monkeys in the study area. The hatching of miracidia from *S. mansoni* eggs in the vervet faeces and establishment of the life cycle of the parasite in the lab-bred mice may suggest that the parasite has zoonotic importance. Nevertheless, molecular studies are required for characterization of *S. mansoni* and other intestinal parasites in humans and monkeys for definite establishment of their zoonotic roles.

## Limitations

The limitations of the present study include examination of a single Kato Ka tz slide per stool sample for schoolchildren and failure to use technique such as modified Ziehl–Neelsen for detection of oocysts of some protozoan parasites in faecal specimens. Attempt was also not made to characterize the genetic structure of the helminth species diagnosed by the Kato Katz and formol-ether concentration techniques in the children and human.

## Abbreviations

NHPs: non-human primates
IRB: Institutional Review Board
